# Direct Testing for Allele-Specific Expression Differences Between Conditions

**DOI:** 10.1534/g3.117.300139

**Published:** 2017-11-22

**Authors:** Luis León-Novelo, Alison R. Gerken, Rita M. Graze, Lauren M. McIntyre, Fabio Marroni

**Affiliations:** * Department of Biostatistics and Data Science, The University of Texas Health Science Center at Houston-School of Public Health, Texas 77030; † United States Department of Agriculture, Agricultural Research Service, Center for Grain and Animal Health Research, Manhattan, Kansas 66502; ‡ Department of Biological Sciences, Auburn University, Alabama 36849; § Genetics Institute and Department of Molecular Genetics and Microbiology, University of Florida, Gainesville, Florida 32603; ** Dipartimento di Scienze Agroalimentari, Ambientali e Animali, Università di Udine, 33100, Italy; †† Istituto di Genomica Applicata, 33100 Udine, Italy

**Keywords:** robust regulation, allelic imbalance, allele-specific expression, gene regulatory networks

## Abstract

Allelic imbalance (AI) indicates the presence of functional variation in *cis* regulatory regions. Detecting *cis* regulatory differences using AI is widespread, yet there is no formal statistical methodology that tests whether AI differs between conditions. Here, we present a novel model and formally test differences in AI across conditions using Bayesian credible intervals. The approach tests AI by environment (G×E) interactions, and can be used to test AI between environments, genotypes, sex, and any other condition. We incorporate bias into the modeling process. Bias is allowed to vary between conditions, making the formulation of the model general. As gene expression affects power for detection of AI, and, as expression may vary between conditions, the model explicitly takes coverage into account. The proposed model has low type I and II error under several scenarios, and is robust to large differences in coverage between conditions. We reanalyze RNA-seq data from a *Drosophila melanogaster* population panel, with F1 genotypes, to compare levels of AI between mated and virgin female flies, and we show that AI × genotype interactions can also be tested. To demonstrate the use of the model to test genetic differences and interactions, a formal test between two F1s was performed, showing the expected 20% difference in AI. The proposed model allows a formal test of G×E and G×G, and reaffirms a previous finding that *cis* regulation is robust between environments.

Regulatory variation can occur within *cis* acting regions (*e.g.*, promoters, enhancers, and other noncoding sequences, directly altering expression of a gene. Other *trans* acting factors such as transcription factors contribute to regulatory variation, but are expected to affect transcription of many target genes with which they interact. By revealing *cis* differences in regulation and controlling for *trans* effects, allelic imbalance (AI) provides a direct window into the relationship between *cis* regulatory sequence and regulation of transcript levels (Brem *et al.* 2002; Yan *et al.* 2002; Lo *et al.* 2003; Wittkopp *et al.* 2004). Testing for regulatory variation that affects expression in *cis* is conceptually straightforward: two alleles within a heterozygote are compared to one another. If AI is observed, there is direct evidence of *cis* differences between alleles; *trans* acting regulation alters expression of both alleles equally, producing an average effect across alleles at a locus, and does not contribute to AI within the heterozygote. Genetic interactions between *trans* acting factors and *cis* regions are expected in some cases, and these interactions are not easily separated from *cis* effects.

*Cis* regulatory differences play a critical role in expression variation within populations (Rockman and Wray 2002; Stranger *et al.* 2012; Graze *et al.* 2014; Fear *et al.* 2016; Wang *et al.* 2017), divergence of gene expression between species (Wittkopp *et al.* 2004; Graze *et al.* 2009; McManus *et al.* 2010), parental imprinting (Crowley *et al.* 2015), and in human health and disease (McCarroll *et al.* 2008; Lin *et al.* 2012; Maurano *et al.* 2012). Importantly, many experimental designs have also incorporated comparisons across different physiological or environmental conditions (von Korff *et al.* 2009; Tung *et al.* 2011; Cubillos *et al.* 2014; Buil *et al.* 2015; Chen *et al.* 2015; Fear *et al.* 2016; Moyerbrailean *et al.* 2016; Knowles *et al.* 2017). *Cis* regulatory differences that are robust to environmental conditions, as well as apparent *cis* variant by environmental interactions, have been reported in these and other studies. However, it is unclear if *cis* variation is similar in most environments, or if there is a significant environmental component of variation in *cis* regulation. Current approaches rely on informal comparisons of AI estimates made separately for each condition, and there is no current formal test for AI by environment interaction.

One inherent, and often neglected, difficulty in measurement of AI is to discriminate real allelic imbalance from bias in estimation of AI, often due to differential mapping of the two alleles on the reference (Degner *et al.* 2009). Early studies of AI used simple designs consisting of two parental strains and a single F1 genotype, with expression measured under standard conditions. These studies often used empirical controls (typically F1 DNA samples) to identify bias in estimation of AI (Wittkopp *et al.* 2004; Graze *et al.* 2009, 2012). However, sequencing DNA for many genotypes, in addition to RNA samples for multiple genotypes and conditions, can be prohibitively expensive. Alternatively, parental DNA reads can be simulated for identification of some forms of bias (Degner *et al.* 2009; Stevenson *et al.* 2013; León-Novelo *et al.* 2014), and/or a range of bias can be examined for the impact on inferences (Fear *et al.* 2016). The use of a strain-specific reference or direct identification of genetic variants from the data also improves the estimation of AI (Skelly *et al.* 2011; Turro *et al.* 2011; Graze *et al.* 2012; Satya *et al.* 2012; León-Novelo *et al.* 2014; Munger *et al.* 2014; Fear *et al.* 2016). These analytical approaches account for bias by filtering regions of likely bias, incorporating information from controls or simulations, or use both filtering and modeling (reviewed in Castel *et al.* 2015).

A variety of approaches have been taken to test for significant allelic imbalance in simple designs (Ronald *et al.* 2005; Degner *et al.* 2009; Zhang and Borevitz 2009; Gregg *et al.* 2010; McManus *et al.* 2010; Rozowsky *et al.* 2011; Skelly *et al.* 2011; Turro *et al.* 2011; Graze *et al.* 2012; León-Novelo *et al.* 2014). Approaches have included linear models for array based studies (Ronald *et al.* 2005; Zhang and Borevitz 2009), binomial or chi-square tests (Degner *et al.* 2009; Gregg *et al.* 2010; McManus *et al.* 2010; Rozowsky *et al.* 2011), and Bayesian models for RNA-seq based studies (Skelly *et al.* 2011; Turro *et al.* 2011; Graze *et al.* 2012; León-Novelo *et al.* 2014). The variance due to random sampling reads from a library can be modeled by treating the total number of reads in a biological replication as a random variable (Graze *et al.* 2012; León-Novelo *et al.* 2014).

For more complicated designs that include multiple genotypes, sexes, and/or environmental conditions, existing studies have primarily used two-step methods, applying an initial model to detect AI in each condition, and then a comparison to determine if AI differs between sample groups. This two-step method has been implemented for a variety of different approaches, including binomial tests with log odds ratios, likelihood ratio tests with Bayesian meta-analysis, and Bayesian models with pairwise Wilcoxon tests (Edsgärd *et al.* 2016; Fear *et al.* 2016; Moyerbrailean *et al.* 2016). For example, as part of their analyses, Fear *et al.* (2016) apply a Bayesian model to determine AI, and identify the genes in AI across 49 test crosses of female *Drosophila melanogaster* flies in only one environment, either virgin or mated. The approach proposed here is more general in the sense that not only does it, as in Fear *et al.* (2016), determines AI in mated and/or in virgin flies, but, in contrast to Fear *et al.* (2016), it also formally tests whether the levels of AI are significantly different in the two environments.

We introduce here a novel Bayesian model (referred to as the environmental model) that allows formal testing of AI between environments, while accounting for potential bias, and model the total number of reads as a random sample from the library. The model can be used with or without empirical control samples to account for bias. In addition, the environmental model explicitly takes into account the expression level of the genic region being examined in each condition, as well as the proportion of reads that are informative for estimating allele-specific expression. The ability to assign reads to the paternal or maternal allele depends upon the amount and location of sequence differences observed between the alleles. The higher the number of differences, and the more even the distribution, the greater the discrimination ability. Lower numbers of differences or large regions with no differences result in greater numbers of unassigned reads that are not informative for estimation of AI. To our knowledge, in all analyses of AI to date, reads uninformative for AI analyses are discarded. The unassigned reads provide us information about the variability of the read counts, and more precise estimate of this variance increases the power to detect AI.

To test the performance of the model with real data, we also conduct a reanalysis of existing data, examining differences in AI for an environmental perturbation in *Drosophila* that has dramatic effects on overall expression, the change from virgin to mated status. During mating, male *D. melanogaster* transfer, together with sperm, a mixture of peptides and proteins into the female reproductive tract; these peptides cause profound changes in female physiology (Zhou *et al.* 2014). As a consequence, mated female flies experience changes in body composition (Everaerts *et al.* 2010), life span (Aigaki and Ohba 1984), and gene expression (Lawniczak and Begun 2004; McGraw *et al.* 2008). Here, we use the environmental model to understand how this large physiological change affects variation in *cis* regulation.

## Methods

### The baseline model

**Figure 1:**
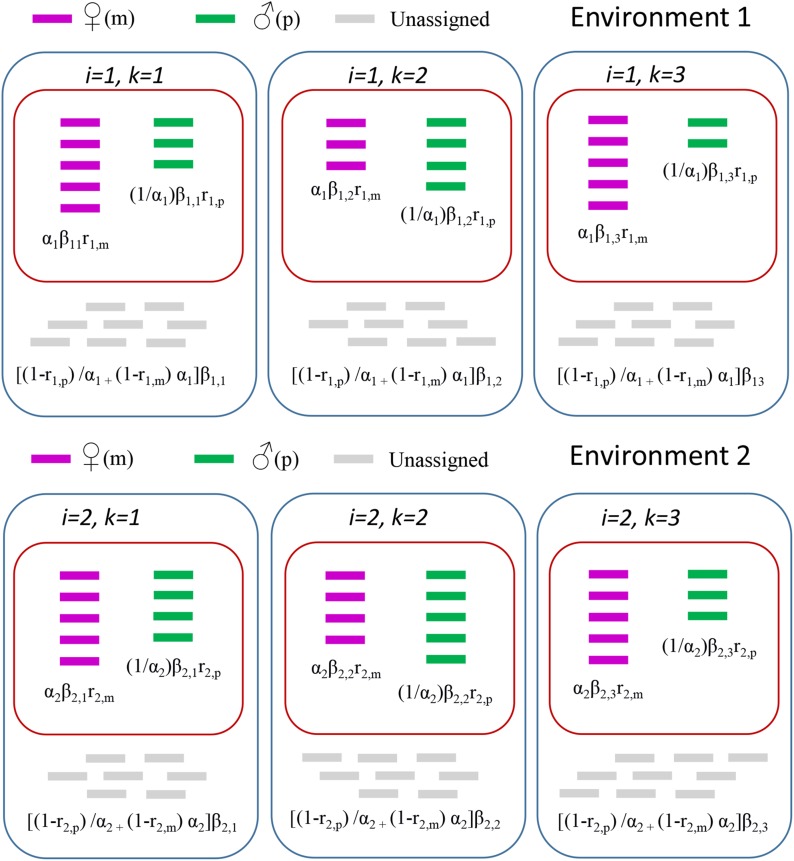
Read assignment. Reads aligning to the maternal allele (*m*) are purple, those aligning to the paternal allele (*p*) are green, and those aligning equally well to both alleles (unassigned) are gray. Expected values according to the environmental model are given in Table 1, and are in black type above. The baseline model uses information of the reads aligning to paternal or maternal alleles only (red boxes), while the environmental model additionally incorporates information about unassigned reads (blue boxes). In the baseline model, μ is the overall expected value, $\beta_{k}$ models the biological replicate variation, α≠1  is equivalent to AI, and the known quantity 1−q is interpreted as the expected proportion of maternal read counts when there is no AI. In other terms, E(x/(y+x)|α=1)=1−q. The priors for all model parameters are gamma(1/2,1/2). The quantities $\sqrt{\alpha }\;\mu \beta_{k}$ and $1/\sqrt{\alpha }$ in the notation of the baseline model play the role of the parameters $\beta_{k}/\left(r_{m}+r_{p}\right)$ and α respectively in the environmental model. When α=1 in the environmental model, $E\left(x|\alpha =1\right)/E\left(x+y|\alpha =1\right)=r_{m}/\left(r_{m}+r_{p}\right).$ This leads us to an interpretation of q under the null in the baseline model as $r_{p}/\left(r_{m}+r_{p}\right)$ in the environmental model.

The baseline model was developed by León-Novelo *et al.* (2014). Briefly, the observed read counts in biological replicate (i) for any single exon/environment are assigned to maternal or paternal alleles, and unassigned reads are discarded (Figure 1). Let $x_{i}$ and $y_{i}$ be the RNA allele-specific read counts from a heterozygote in biological replicate i(i = 1, … I):

$$\begin{array}{l} y_{i}|\mu ,\alpha ,\beta_{i},q∼\text{Poisson}\left(\mu \alpha \beta_{i}q\right),\text{and}\\ x_{i}|\mu ,\beta_{i},q∼\text{Poisson}\left(\mu \beta_{i}\left(1-q\right)\right). \end{array}$$

Here, μ is the overall mean, $\beta_{i}$ is the variation of biological replicates (i=1, …, I) due to the random sampling of reads from libraries, α is the effect of a read having AI, and q incorporates bias information, where values >0.5 indicates bias toward the *y* allele, and values <0.5 is bias toward the *x* allele. If θ is the real proportion of reads from the *y* allele then:

$$\theta =\frac{\mu \alpha \beta_{i}}{\mu \beta_{i}+\mu \alpha \beta_{i}}=\frac{\alpha }{1+\alpha },$$

when there is no AI (θ=0.5), therefore α=1. The bias correction parameter is *q*; it can be a random variable estimated from DNA controls, from simulation, or a fixed constant varied to reflect uncertainty (Fear *et al.* 2016).

### The environmental model

Let *i* = 1,2 be the index of condition, which can represent environmental differences or differences in treatments (*e.g.*, mated and virgin status) or differences between genotypes. We define them as environment 1 and environment 2.

Assume that we have *K*_1_ biological replicates for environment 1 and *K*_2_ replicates for environment 2.

For each gene/gene region to be examined, we define:

*x_i_*,*_k_* = number of RNA reads aligned to the “maternal” allele in environment *i* and replicate *k*,
*y_i_*,*_k_* = number of RNA reads aligned to the paternal allele.
*z_i_*,*_k_* = number of RNA reads aligned equally well to both alleles, and that cannot be assigned to either allele; they are also referred to as “unassigned”; and
*r_i_*,*_p_* (*r_i_*,*_m_*) = probability that a read generated from paternal (maternal) aligns to paternal.
(maternal) for environment *i*. In specific notation:
r*_i_*,*_p_* = P*r*[read aligns to paternal|read generated by paternal] for *i* = 1,2.
1−*r_i_*,*_p_* = Pr[read is unassigned|read generated by paternal] for the genotype in environment *i*.

*r_i_*,*_p_* (*r_i_*,*_m_*) can be estimated from control DNA or from simulation. To estimate *r_i_*,*_p_*, (1) use the paternal genome to simulate all possible reads that can be generated by the paternal allele, (2) align these reads to both the paternal and maternal genomes, and (3) estimate *r_i_*,*_p_* as the proportion of the simulated reads aligning best to the paternal genome. Note that the true proportion of simulated reads aligning best to the parental allele in the two environments (*r_1_*,*_p_* and *r_2_*,*_p_*) for the same genotype is expected to be similar. However, to allow for comparisons across genotypes where this assumption may not hold, the model does not force these two parameters to be identical.

We assume that the distribution of the counts *x_i_*,*_k_*; *y_i_*,*_k_*; *z_i_*,*_k_|α_i_*, *β_i_*,*_k_* have the expected values given in Table 1. A graphical representation of the expected values is provided in Figure 1. The biological replication specific random effect *β_i_*,*_k_* corrects for the random sampling of reads from the library for each biological replication. As with the baseline model, we assume that the total number of reads from a biological replication is a random effect with expected value $\beta_{i,k}\left(\alpha_{i}+1/\alpha_{i}\right).$ This explicitly recognizes the sampling of material from a library in order to generate the observed reads and incorporates the resulting sampling variance directly into the model. Approaches that consider the total number of reads in a biological replication to be fixed often fail to model the overdispersion of the data. Allowing it to be random allows modeling of the overdispersion.

**Table 1:** Expected values for read counts under the proposed model for environment *i* and replicate (*i*, *k*), where *i* = 1, 2 and *k* = 1,2*….k_i_*

Maternal (*x_i_*,*_k_*)	Paternal (*y_i_*,*_k_*)	Unassigned (*z_i_*,*_k_*)
*α_i_β_i_*,*_k_r_i_*,*_m_*	(1/*α*_i_)*β_i_*,*_k_r_i_*,*_p_*	[(1−*r_i_*,*_p_*)/*α_i_*_+_ (1−*r_i_*,*_m_*) *α_i_*]*β_i_*,*_k_*

Every replicate produces three observed counts for each gene/gene region: one for reads aligning better to the maternal allele, one for reads aligning better to the paternal allele, and one for reads aligning equally well to both alleles, and therefore unassigned to either the paternal or maternal allele. The sum of the expected counts will be the expected total number of reads aligned to that gene/gene region and an estimate of overall expression. Note that the number of reads is considered random to account for variation due to sampling reads from the library.

For example, the maternal read count of the third virgin replicate, $x_{2,3},$ has an expectation of $\alpha_{2}\beta_{2,3}r_{2,m}.$ Note that when *r_i_*,*_p_* and *r_i_*,*_m_* are both equal to 1, we expect all the reads to be assigned to an allele, and the proportion unassigned to be zero (*i.e.*, *z_i_*,*_k_* = 0). The proportion of reads assigned to the maternal (paternal) allele compared to those that are not assigned will depend on several factors, such as read length, the level of divergence between the two haplotypes, the alignment algorithm, and the stringency of alignment. These parameters are not equal to reflect the possibility that the quality of the genotype specific references may not be equivalent between the maternal and paternal reference, and/or that for a particular gene/gene region there may be unobserved structural variation in one but not both alleles. DNA controls can account for factors such as hidden structural variation, but can be prohibitively expensive. In the absence of DNA controls, the use of simulations can estimate some of the potential bias, and inclusion of these estimates in the model is preferable to ignoring sequence and mapping bias altogether (León-Novelo *et al.* 2014).

*β_i_*,*_k_/α_i_* and *α_i_β_i_*,*_k_* are the total expected number of reads from the paternal and maternal alleles, respectively, in environment *i* and biological replication *k*. Of the *β_i_*,*_k_/α_i_* reads coming from the paternal allele, we expect (1/*α_i_*)*β_i_*,*_k_r_i_*,*_p_* to be assigned to the paternal allele, and (1−*r_i_*,*_p_*)*β_i_*,*_k_/α_i_* to be unassigned. Similarly, of the *α_i_β_i_*,*_k_* reads from the maternal allele, we expect *α_i_β_i_*,*_k_r_i_*,*_m_* to be assigned to the maternal allele and (1−*r_i_*,*_m_*)*α_i_β_i_*,*_k_* to be unassigned. The parameter $\alpha_{i}^{2}$ is the ratio of the maternal and paternal expected value counts for genotype *i*, after adjusting for assignment bias. Explicitly allowing for reads to be unassigned allows the level of expression to be included in the modeling.

The notation is the following:

$$\alpha_{i}^{2}=\frac{E\left(x_{i,k}\right)/r_{i,m}}{E\left(y_{i,k}\right)/r_{i,p}},$$

for *i* = 1,2 and *k* = 1,2,…,*K_i_*.

We are interested in testing the null hypotheses:

1. Allelic balance in environment 1 (*e.g.*, mated) or, equivalently, H_01_: *α*_1_ = 1.
2. Allelic balance in environment 2 (*e.g.*, virgin), H_02_: *α*_2_ = 1.
3. The level of AI is the same in both (or independent of) environments. Equivalently, H_03_: *α*_1_ = *α*_2._

Notice that H_03_ is testing if the true proportion of reads coming from the paternal allele in environment 1 (*e.g.*, mated) is the same as in environment 2 (*e.g.*, virgin), or, in other words, the level of AI is the same in both conditions (*e.g.*, mated and virgin). This is a formal test for the interaction between AI and environment.

The proportion of counts coming from the maternal allele (line) in environment i is then:

$$\theta_{i}=\frac{E\left(x_{i,k}/r_{i,m}\right)}{E\left(x_{i,k}/r_{i,m}+y_{i,k}/r_{i,p}\right)}=\frac{\alpha_{i}}{\alpha_{i}+1/\alpha_{i}}.$$

$\theta_{i}$ is expected to be 0 when no counts are coming from the maternal allele, 1 when all the counts are coming from the maternal allele, and 0.5 in the case of perfect allelic balance. The model is implemented in R (R Core Team 2017), and is available as Supplemental Material, File S2.

### Negative binomial sampling model

Above, we describe the model for the expected value counts, and now we model the counts. We assume the counts follow a negative binomial distribution, with expected values given in Table 1. For example, the distribution for the read counts in the third virgin biological replicate aligning to the maternal allele is:

$$\;x_{2,3}∼NB\left(\text{mean}=r_{2m}\beta_{2,3}\alpha_{2},\text{dispersion}=\varphi \right).$$

The dispersion parameter φ is common to the distribution of all read counts. Here x∼NB(μ,φ) denotes the negative binomial distribution with mean μ and variance $\mu +\varphi \mu^{2}.$ RNA-Seq data typically exhibit overdispersion (with respect to the Poisson model; *i.e.*, the variance is greater than the mean). Since the negative binomial sampling distribution can model overdispersed data, it has been proposed as a sampling distribution for RNA counts (Robinson and Smyth 2007; Anders and Huber 2010; Di *et al.* 2011; León-Novelo *et al.* 2017).

To complete the Bayesian model, we specify the prior distributions for the model parameters:

$$\alpha_{1},\alpha_{2}∼lognormal\;\left(\mu_{\alpha }=0,\sigma_{\alpha }^{2}=1/4^{2}\right)$$

$$\beta_{1,1}\;,\ldots ,\beta_{1,K_{1}},\beta_{2,1},\ldots ,\beta_{2,K_{2}}∼gamma\left(a_{\beta }={\tilde{a}}_{\beta },b_{\beta }\right)$$

$$b_{\beta }∼gamma\left(a_{b_{\beta }}=2,b_{b_{\beta }}=2\;{\tilde{b}}_{\beta }\right)$$

$$\varphi ∼Inverse-gamma\;\left(a_{\varphi }=2.01,b_{\varphi }=0.05\right).$$

Here, gamma(a,b)denotes the gamma distribution with mean shape and rate parameters a and b.

The computation of credible intervals for the parameters of interest $\alpha_{1},\alpha_{2}$ and $\alpha_{1}/\alpha_{2}$ is carried out using Markov Chain Monte Carlo. We reject H_01_ when the credible interval for $\alpha_{1}$does not contain 1. Similarly, we reject H_02_ (or H_03_) when the credible interval for $\alpha_{2}$ (or $\alpha_{1}/\alpha_{2})$ does not contain 1.

The lognormal prior distributions for the parameters $\alpha_{1}$and $\alpha_{2}$ were used to make the model symmetric with respect to the labels paternal and maternal (in our example, tester and line, respectively). This is *a priori* $\alpha_{i}∼1/\alpha_{i}$ so the estimates of $\alpha_{i}$ in the baseline model are the same as the estimates of $1/\alpha_{i}$ in the model swapping the labels of tester and line. The hyper-parameter $\sigma_{\alpha }^{2}=1/4^{2}$ is chosen so that the prior expected value of $\alpha_{i}$ equals $\mu_{\alpha }+exp\left(\sigma_{\alpha }^{2}/2\right)=1.03\approx 1,$ and prior variance $\left(exp\left(\sigma_{\alpha }^{2}\right)-1\right)\times exp\left(2\mu_{\alpha }+\sigma_{\alpha }^{2}\right)=0.4168,$ so, in the case of noninformative data (*e.g.*, very small counts), the posterior distribution of $\alpha_{i}$ is similar to the prior, and we do not conclude AI. The gamma prior for $b_{\beta }$ “tightens” the $\beta_{ik}$s to be similar.

Setting a prior in the hyper-parameters of the prior distribution of $\beta_{ik}$s allows a “borrow of strength” across different biological replicates. To set the hyper-parameters of the prior distribution for $b_{\beta },$ we use an “empirical Bayes approach.” First, we obtain (rough) estimates for $\beta_{ik}$s. Second, we assume that they follow a gamma distribution, and compute the MLE estimates ${\hat{a}}_{\beta }$ and ${\hat{b}}_{\beta }$ of the shape and rate parameters, respectively. Third, we set the parameter in the model $a_{\beta }\equiv {\hat{a}}_{\beta },$ and we set $a_{b_{\beta }}=2{\hat{b}}_{\beta }$ and $b_{b_{\beta }}=2$ so that $E\left(b_{\beta }\right)={\hat{b}}_{\beta }$ and $var\left(b_{\beta }\right)\;=2{\hat{b}}_{\beta }.$ Since we have few data points to estimate φ, we use an informative prior with mean $b_{\varphi }\left(a_{\varphi }-1\right)\approx 0.05$ prior with variance $b_{\varphi }^{2}/\left[{\left(a_{\varphi }-1\right)}^{2}\left(a_{\varphi }-2\right)\right]\approx 0.05.$

### Measuring type I and II error rate

Read counts were simulated according to a negative binomial distribution in several different scenarios. In each scenario, read counts were simulated for 1000 gene regions (for convenience, we refer to these as exons) independently in two different environments, with three replicates per environment. The number of reads mapping in each exon was simulated according to a negative binomial distribution with size = 50 and expected value equal to *β_i_*,*_k_* × *η*, where *β_i_*,*_k_* is the effect of biological replicate in environment *i* and replicate *k*, and *η* is a factor used to simulate different levels of gene expression (and/or sequencing coverage), which in turn affect the power of detecting AI. Simulations were run with *η* = 10 (low expression/coverage) and *η* = 100 (high expression/coverage). *β_i_*,*_k_* was varied as shown in Table 2.

**Table 2:** Values of β used in the simulations

	*k = 1*	*k = 2*	*k = 3*
*i = 1*	1	1.2	0.7
*i = 2*	1	1.3	0.8

Different levels of AI in the environment 2 were simulated for sets of 1000 exons by varying *α*_2_ from 0.5 to 2. No AI was simulated in environment 1 (*i.e.*, *α*_1_ = 1); 11 different levels of log2 of the ratio α_1_/α_2_ were simulated, varying from −1 and 1 with step 0.2.

Read counts were simulated without bias, *i.e.*, with *r_p_* = *r_m_*. To assess robustness of the model to misspecification of bias, unbiased simulated counts were fed to the model, and analysis of AI was conducted after providing biased prior estimates of *r_p_* and *r_m_*.

The bias in estimation of AI is not always correctly captured by simulation or by DNA controls. This means that, even when controls are used, bias may be mis-specified. It is important to understand how the model performs with different levels of mis-specification. Bias mis-specification was measured as *x* = 2(100)(*r*−1), where (*r*−1) is the difference between the mis-specified value of *r_p_* (or *r_m_*) used to fit the model, and the simulated value of *r_p_* (or *r_m_*), *i.e.*, 1. Nine different levels of mis-specification *x* were used: −40, −30, −20, −10, 0, 10, 20, 30, and 40%.

When maternal and paternal alleles have similar sequences in the coding region, the proportion of reads aligning equally well to both alleles is high. A series of simulations was conducted using the expected number of unassigned reads *z_i_*,*_k_*. To assess the effect of variation in the proportion of unassigned reads, an additional series of simulations was conducted by multiplying the expected values of *z_i_*,*_k_* by 10,000. We will refer to these two scenarios as “unassigned inflation factor = 1” and “unassigned inflation factor = 10,000,” respectively.

The first scenario can be observed when the paternal and maternal haplotypes are different, and most reads can be assigned. The second scenario is observed when most of the reads cannot be assigned to either haplotype, and is used as a worst case scenario of very similar paternal and maternal haplotypes. In any given experiment, we expect some genes to follow each of these two scenarios.

To measure the robustness of the model to unequal gene expression across conditions, we repeated the simulations comparing one condition with high coverage, and one with low coverage, *i.e.*, we run a simulation setting *η*_1_ = 10 and *η*_2_ = 100, and a simulation setting *η*_1_ = 100 and *η*_2_ = 10. This is an important consideration as differential expression should not be confused with AI.

Type I error was assessed using simulations in which *α*_1_ = 1 and *α*_2_ = 1 (*i.e.*, no AI in either environment). Type II error of AI detection, and of the detection of difference in AI across environments, was assessed using simulations in which *α*_1_ = 1 and *α*_2_ ≠ 1.

### Reanalysis of D. melanogaster data (mated *vs.* virgin)

#### Data retrieval and cleaning:

RNA-seq data from a panel of 68 *D. melanogaster* F1 hybrids obtained from crossing 68 strains with the w1118 laboratory strain (Kurmangaliyev *et al.* 2015) were used to measure allele specific expression. Mapping of reads on genotype-specific references and quantification of reads aligning to each exon was performed as previously described (Fear *et al.* 2016). Briefly, reads originating from each cross were aligned on the strain-specific references (obtained from FlyBase v5.51) for the two parental genomes, and reads were classified as aligning in paternal (tester), maternal (ine), or unassigned (when reads mapped equally well on both references).

Based on simulations, low numbers of reads assigned to maternal or paternal allele, and/or high numbers of reads that cannot be assigned to either allele lead to inflated type I error. For this reason, instances in which the proportion of total reads assigned to either parental allele was <1% (*i.e.*, having either very low number of assigned reads or very high number of unassigned reads) were excluded from analysis.

Analysis was performed on a total of 169,842 data points (termed “exons × lines,” *i.e.*, the number of exons across all lines), belonging to 13,898 different exons in 68 crosses. In the manuscript, the terms “data point” and “exons × lines” will be used to indicate the sum of informative lines across all the informative exons (or equivalently, the sum of informative exons across all the informative lines), as follows:

$$\text{Data}\;\text{points}\left(\text{exons}\times \text{lines}\right)=\;\sum_{i=1}^{n}\sum_{j=1}^{m}e_{i}l_{j},$$

where *e_i_* and *l_j_* are 1 if exon *i* in line *j* is informative and zero otherwise, and *n* and *m* are the total number of exons and lines considered, respectively.

For the purpose of analysis, data were aggregated by (a) exon, (b) line, or (c) exon in line. We detail below the different meanings. One gene composed of two exons, one with data in 10 crosses, and one with data in 15 crosses, respectively, has information in two exons, in 12.5 lines (average) and 25 data points. A gene consisting of 25 exons, all with data in just one cross, is said to have information in 25 exons, one line, and 25 data points.

#### Comparing the environmental model with the baseline model:

A Bayesian model that accounts for bias (León-Novelo *et al.* 2014) has been already applied to the *D. melanogaster* data presented here (Fear *et al.* 2016); we will refer to it as the “baseline” model. The model presented here extends the baseline model significantly, and directly compares AI between environments; it will be referred to as the “environmental” (as opposed to the “baseline”) model. Results were compared for a total of 66,504 data points that passed quality control in both the previous (Fear *et al.* 2016) and present study. Pearson’s correlation coefficients between the posterior estimates of the proportion of reads aligning on the paternal allele (ϑ) were estimated for mated and virgin flies according to results of the baseline and environmental model, respectively.

To determine the effect of coverage (number of reads mapped on each exon) on AI in the baseline and environmental model, exons were stratified into four groups: (1) no AI according to either model, (2) AI according to the environmental model only, (3) AI according to the baseline model only, and (4) AI in both models. Mean coverage was assessed by measuring the mean number of reads mapping an allele specifically in each exon in mated and virgin lines. The distribution of coverage among exons without AI was compared to the distribution of coverage in the remaining three classes using the Wilcoxon-Mann-Whitney test, separately for mated and virgin flies. The distribution of the deviation of ϑ from the expected value of 0.5 was plotted in exons showing, or not showing, AI in either model in virgin and mated flies, separately. The distribution of the differences in the estimate of ϑ between the two models were plotted, stratifying AI classification according to the two models: no AI, AI according to either one of the models, AI according to both models. Statistical analysis were performed in R (R Core Team 2017).

#### Analysis of AI in mated and virgin flies using the environmental model:

The RNA-seq data included in this study has been described before (Kurmangaliyev *et al.* 2015). Briefly, RNA was obtained from F1 mated and virgin flies originated by crossing females of each of 68 lines with mates of a common tester (w1118). Data points were included in the study if at least 50 reads could be mapped to either paternal or maternal allele in both virgin and mated flies, and if at least 200 total reads were mapped to the exon (irrespectively of them being assigned to the paternal or maternal allele, or being unassigned). The environmental model was used to simultaneously estimate AI in mated female flies, in virgin female flies, and to test for AI by environment interaction (G×E). In total, 169,842 data points were analyzed.

As each of the F1 genotypes has been evaluated for AI, it is of interest to assess the population frequency of AI in any given exon, Fisher’s exact test was performed on a 2 × 2 contingency table, reporting for each exon and the number of F1 genotypes showing different levels of AI between conditions and those not showing differences in AI between conditions. Exons showing higher (or lower) than expected frequency of AI between conditions were identified by testing the hypothesis that the odds ratio in the contingency table is >1 or <1.

A further demonstrative analysis of how genotypes may be compared across the population was performed by explicitly comparing AI in two different F1 genotypes, treating each F1 genotype as an environment. The environmental model was used to simultaneously estimate AI in r365×w1118 F1’s, in r907×w1118 F1’s, and to test for AI by F1 interaction (G×G).

### Data availability

Sequences used for the present work were retrieved from SRA under the accession number PRJNA281652. Detailed description of the procedures for obtaining F1 genotypes is available at https://github.com/McIntyre-Lab/papers/tree/master/lehmann_2015/original_data. File S1 includes supplemental methods, Figures S1–S5 in File S1 with legends, and Table S1 in File S1 with legend. The supplemental material includes an implementation of the model in R and a toy data set. Instructions are provided in File S2.

## Results and Discussion

### Measuring type I and II error rate of the environmental model

We assessed type I and II error simulating various scenarios that could be experienced in real experiments. We varied *η*, a proxy of the number of reads mapped to each feature, and thus representative of the combination of gene expression and average sequencing depth (coverage). In addition, we varied the proportion of reads that cannot be assigned to either allele (unassignment). High unassignment rates can be observed when the paternal and maternal allele are similar, and a large proportion of reads cannot be unambiguously attributed to either allele.

**Figure 2:**
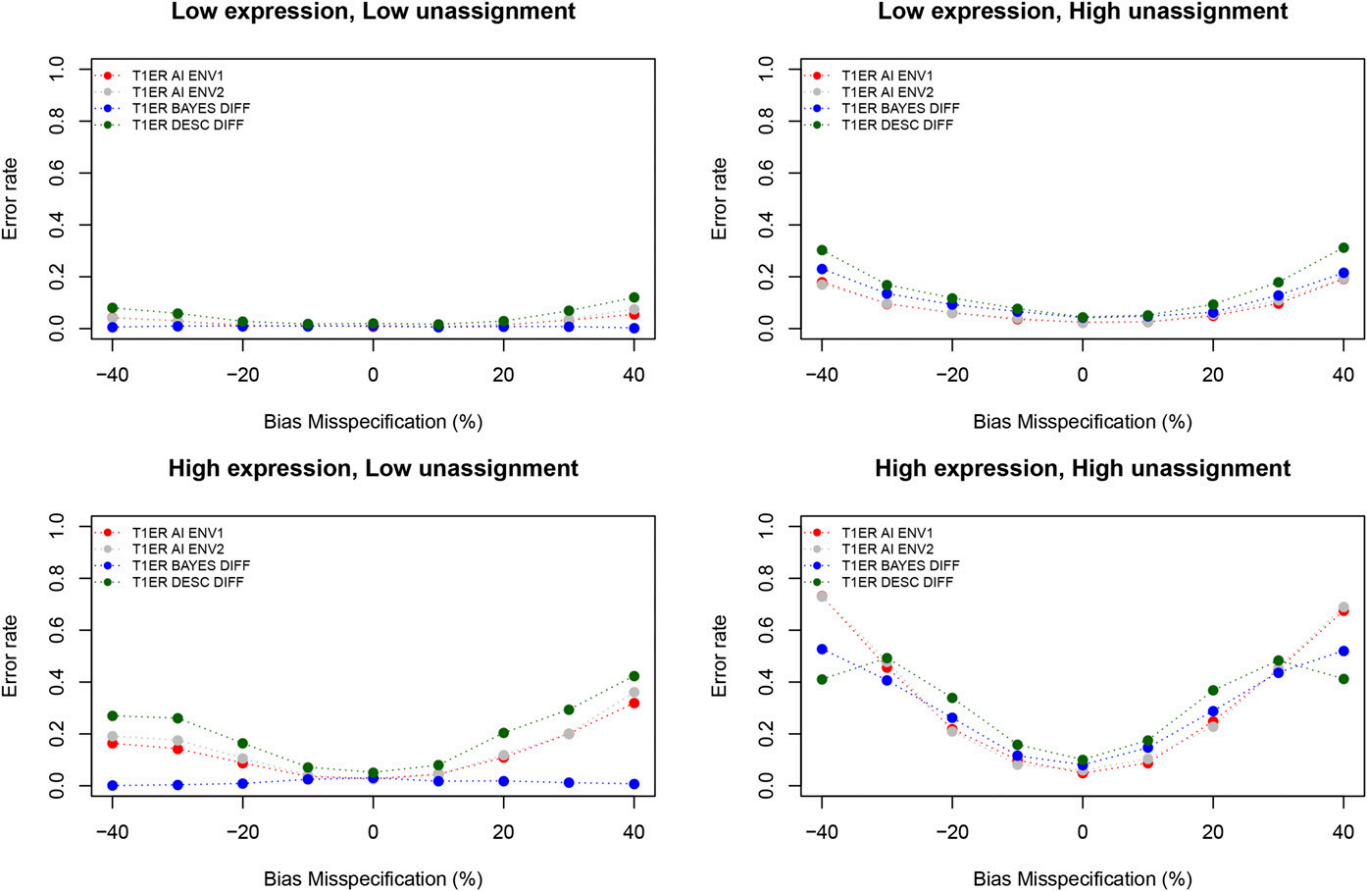
Type I error rate. The two upper panels show Type I error rate in the case of low gene expression, and the two bottom panels show Type I error rate in the case of high gene expression. Panels on the left show results obtained by specifying the number of reads aligning equally well to both alleles as expected under the model in Table 1. Panels on the right shows results obtained by multiplying that quantity by 10,000. We plot in red the type I error rate in environment 1, in gray the type I error rate in environment 2, and in blue and green the type I error rate in detecting different levels of AI between environments using the Bayesian approach implemented in the environmental model, and the descriptive method as implemented in the baseline model, respectively. Low expression: *η* = 10; High Expression: *η* = 100; Low unassignment: unassigned reads = *z_i_*,*_k_*; High unassignment: unassigned reads = 10,000 × *z_i_*,*_k_*

Results shown in Figure 2 report Type 1 error rate as a function of bias misspecification at two different levels of gene expression (or coverage), and two different levels of unassigned reads. Unlike the baseline model, the type I error rate when the number of unassigned reads follows the model expectation (Table 1), and the bias is not higher than previously reported in interspecific crosses, where it has been measured by DNA (Graze *et al.* 2012; León-Novelo *et al.* 2014), is close to the expected nominal levels.

Only when the number of unassigned reads is 10,000 times the expected value (Table 1), do type I error rates climb. As expected, when bias is high, and unaccounted for in the model, the type I error rate can be very high. Under this “worst case” scenario, the environmental model still has lower type I error relative to the baseline model.

When the number of unassigned reads matches the model expectation (Table 1), the type I error rate for detecting differences in AI across environments (blue lines and dots in Figure 2) is <5% for all bias misspecification levels.

**Figure 3:**
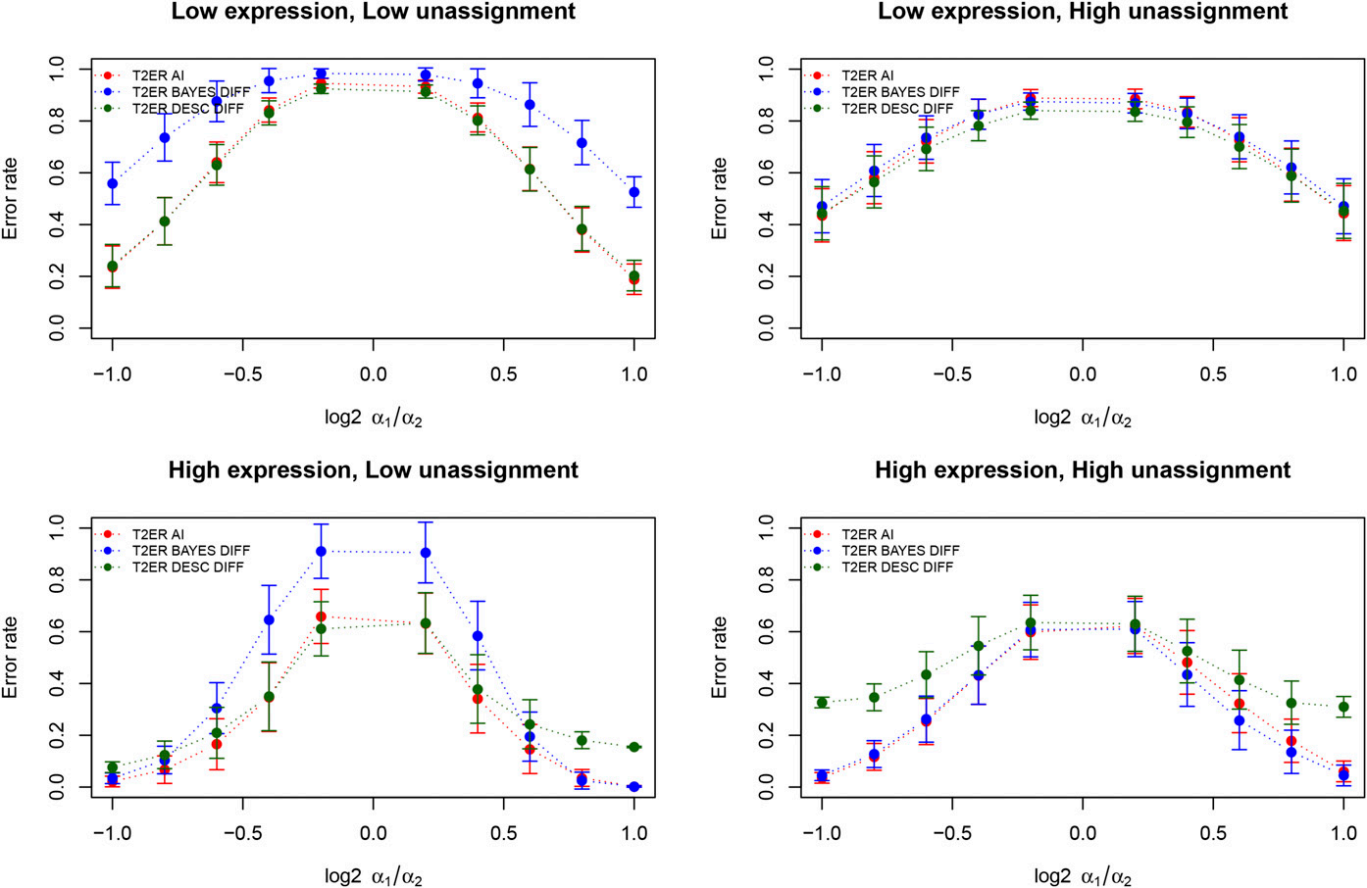
Type II error rate. Type II error rate when comparing an environment for which no AI was present (*α*_1_ = 1) with an environment with varying levels of AI. Differences in AI between environments are reported as log2 *α*_1_/*α*_2_. The two upper panels show Type II error rate in case of low gene expression, and the two bottom panels show Type II error rate in case of high gene expression. Panels on the left show results obtained by specifying the number of reads aligning equally well to both alleles as expected under the model in Table 1. Panels on the right shows results obtained by multiplying that quantity by 10,000. We plot in red the type II error rate in environment 1, in blue the type II error rate for difference in AI between environments, and in green the type II error rate between the environments when using the descriptive approach as implemented in the baseline model. Each point represents the average over the seven simulated levels of bias misspecification, and the error bars represent SE. Low expression: *η* = 10; High Expression: *η* = 100; Low unassignment: unassigned reads = *z_i_*,*_k_*; High unassignment: unassigned reads = 10,000 × *z_i_*,*_k_*

As expected, the type II error rate increases when log2 *α*_1_/*α*_2_ approaches zero. At each value of log2 *α*_1_/*α*_2_, lower expression leads to a higher type II error rate. The effect of the misspecification of the proportion of unassigned reads on type II error is small (Figure 3).

Further analyses were performed simulating 10-fold difference in coverage between conditions. When higher coverage occurs in environment 2 (Figure S1 in File S1 left panel), type I error in environment 2 is higher, and vice versa (Figure S1 in File S1 right panel). The detection of differences in AI between environments might be negatively affected by different levels of coverage across the experiments. Our simulations show that in this case, as previously reported, the baseline model has a high type I error while the environmental model has very low type I error (Figure S1 in File S1). This suggests that the environmental model does, as expected, protect from type I error under different coverage levels. Correspondingly, the Type II error is higher for the environmental model compared to the baseline model. This is especially true when the difference in α is small (Figure S2 in File S1).

### Reanalysis of D. melanogaster data

#### Comparing the environmental model with the baseline model:

We provide here a comparison of the results from the environmental model to those published using the baseline model (Fear *et al.* 2016), for analysis of differences in AI across mated and virgin physiological states and F1 genotypes. A total of 62,448 data points (4880 different exons and 49 different lines) were included in the final analysis. On average, each exon had detectable expression in 14 lines (ranging from 1 to 49 detected), and each line contains 1375 exons (ranging from 446 to 2832 detected). Figures S3 and S4 in File S1 show, for each of the 49 lines, the proportion of exons flagged with AI in mated and virgin flies, respectively. The figures suggest that the two models have similar behavior in the analyzed lines, with the environmental model being generally more conservative and showing less extreme differences across lines. According to the environmental model, line r907 has the highest proportion of exons with AI both in mated (44%) and virgin flies (44%). According to the baseline model, line r907 has the highest proportion of exons with AI in mated flies (42%), and line r502 has the highest proportion of exons with AI in virgin flies (46%).

**Figure 4:**
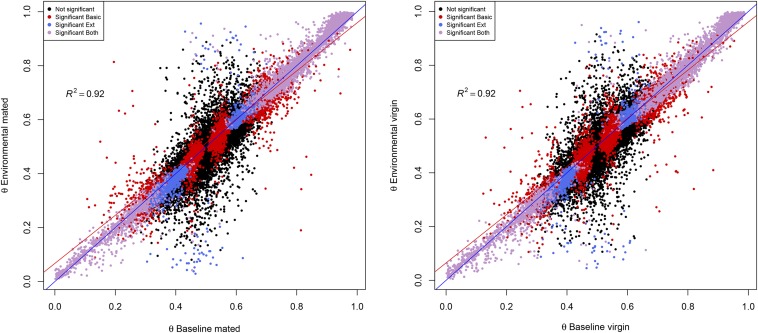
Posterior estimate of ϑ. Posterior estimate of ϑ according to the baseline and environmental model in mated (left) and virgin (right) flies, respectively. Each dot represents a data point. Black points: no AI in either set. Red Points: AI detected by baseline model alone. Blue points: AI detected by environmental model alone. Purple points: AI detected by both models. Blue line: bisector of the first quadrant angle. Red line: regression line.

Table 3 reports the number of data points showing AI in mated and virgin flies, according to the baseline and environmental models, respectively. In general, the baseline model is less conservative than the environmental model, detecting 12,330 and 12,581 events of AI in mated and virgin flies, respectively, *vs.* 10,051 and 10,133 detected by the environmental model. Cohen’s kappa (Cohen 1960) between the two models is 0.49 in both mated and virgin flies. Posterior estimates of ϑ were strongly correlated between the two models, with *R*^2^ of 0.92 in both mated and virgin flies (Figure 4).

**Table 3:** Number of data points (exons × lines) showing AI

Mated	Environmental *α* = 1	Environmental *α* ≠ 1
Baseline *α* = 1	46,544	3574
Baseline *α* ≠ 1	5853	6477
Virgin	Environmental *α* = 1	Environmental *α* ≠ 1
Baseline *α* = 1	46,353	3514
Baseline *α* ≠ 1	5962	6619

**Figure 5:**
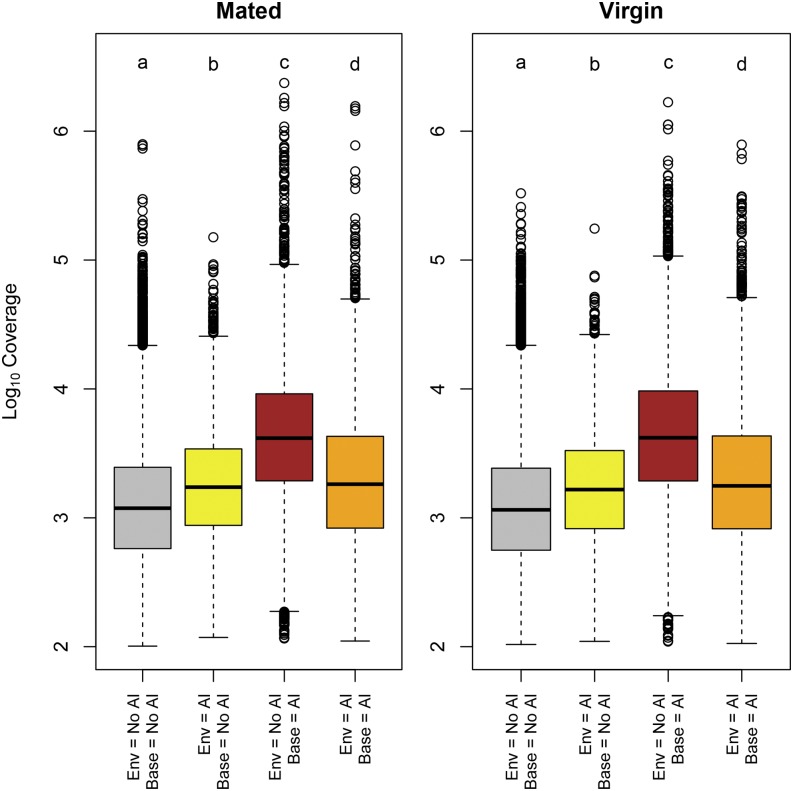
Exon coverage and AI. Base 10 logarithm of coverage of exons for which no model detected AI (gray boxes), only the environmental model detected AI (yellow boxes), only the baseline model detected AI (dark red boxes), or both models detected AI (orange boxes), respectively.

We compared coverage of exons stratified by significance according to either environmental or baseline model. According to Wilcoxon’s test, coverage in exons not showing AI in either model was significantly lower than all other classes (Figure 5). The result is expected, since genes with low coverage are expected to provide lower power for the detection of AI (León-Novelo *et al.* 2014). Exons showing AI only according to the baseline model had higher coverage than all the remaining categories. This might be due to false positives in the baseline model. Simulation results showed that the power of the environmental model increases as coverage increases, with a relatively constant type I error rate, while the baseline model has an increase in type I error at higher coverage. This is likely due to the inclusion of the unassigned reads as an estimate of coverage in the environmental model. The coverage of exons for which only the environmental model showed AI and of exons for which both models detected AI, did not differ significantly.

**Figure 6:**
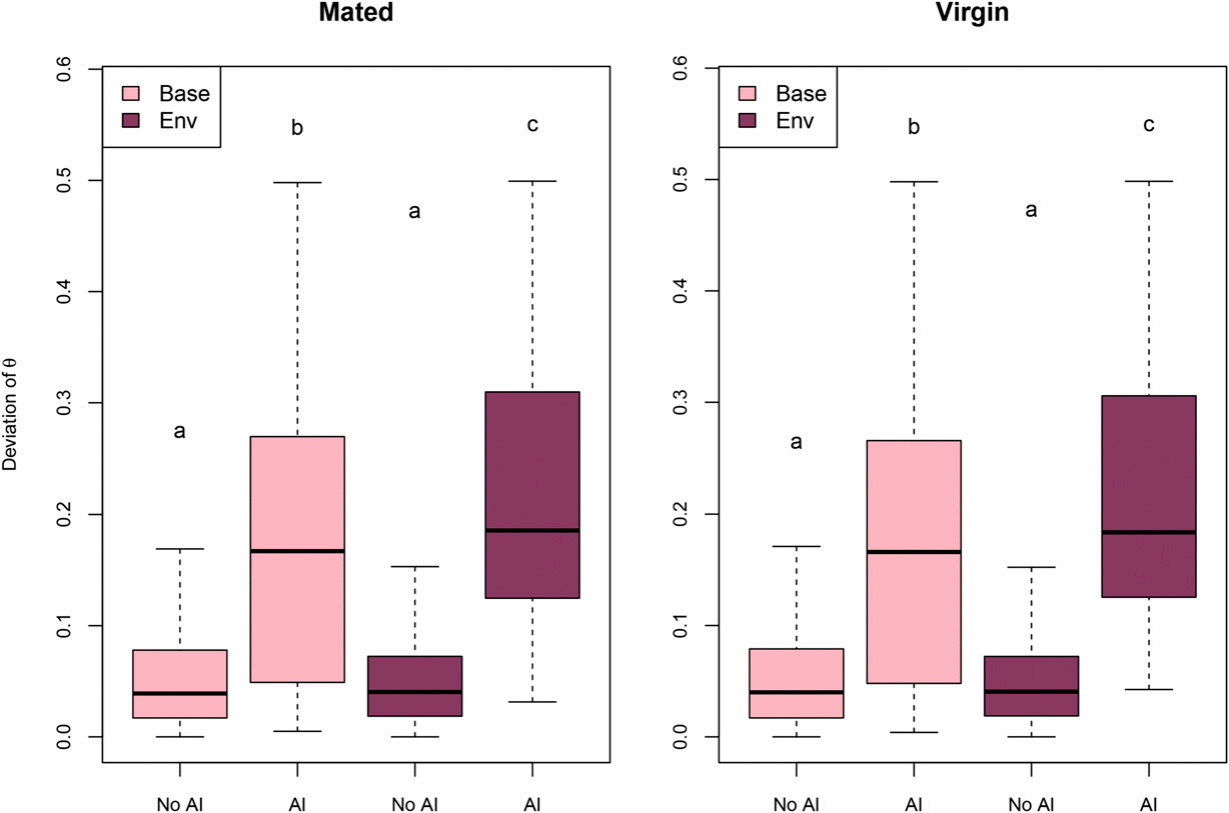
Deviation from expected ϑ and AI. Distribution of the absolute value for the deviation of ϑ from the expected value of 0.5 in exons for which AI was detected (*α* = 1) or not (*α* ≠ 1), according to the baseline (light pink) or environmental model (dark red). Differences between groups according to Wilcoxon pairwise test are shown. Groups sharing a letter are not different. Groups not sharing letters are different.

As expected, the estimated deviation in AI from the null expectation is greater in exons for which AI was detected in both models (Figure 6). In addition, exons in which AI was detected by the environmental model showed higher median deviation than exons for which AI was detected by the baseline model. No significant difference between baseline and environmental model was observed for the estimates of ϑ in exons without AI.

**Figure 7:**
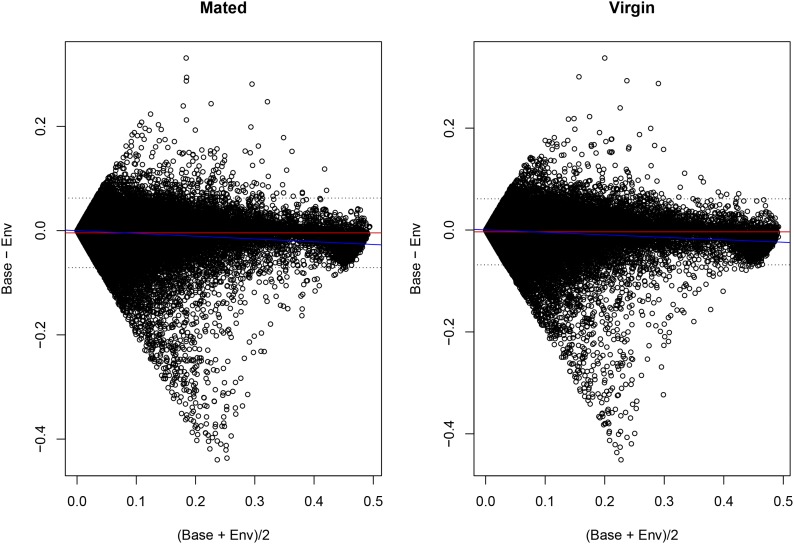
BA plot. BA plot comparing estimates of ϑ according to the baseline (Base) and environmental (Env) model in mated and virgin flies, respectively. The red line represents the mean difference. The dotted lines are the 95% confidence intervals of the difference. The regression of the difference over the mean is represented by the blue line.

Figure 7 shows the BA plot comparing the two methods (Altman and Bland 1983). The plot shows that a minority of data points elicit different estimates of ϑ between baseline and the environmental model, especially for intermediate estimates of ϑ. We compared the number of data points for which differential AI between conditions was detected by the environmental and baseline models stratifying by discrepancy in ϑ estimates (Table S1 in File S1). Data points with a difference in ϑ estimate (*i.e.*, those lying outside of the 95% confidence interval) showed stronger disagreement between the environmental and the baseline model in detecting differential AI across conditions (Fisher’s exact test p-value = 7.01E−07 for data stratified by difference in ϑ estimates in mated flies, and p-value = 2.27E−06 for data stratified by differences in virgin flies). The difference is mainly due to instances in which the baseline model detects differences in AI and the environmental model does not (p-value = 2.62E−07 and p-value = 1.79E−05 for estimates of ϑ in mated and virgin flies, respectively). Instances in which the environmental model alone detects differential AI between conditions do not vary between points lying inside or outside of the confidence intervals (p-value = 0.87 and p-value = 0.07 for estimates of ϑ in mated and virgin flies, respectively). This means that when the estimates of ϑ differ, the baseline model shows a smaller difference in AI across conditions.

The baseline model uses a descriptive approach to consider AI differences in mating status, by which an exon is classified as showing different AI values in the two statuses, if and only if, it shows AI only in one status. This descriptive approach can be applied to the environmental model (in addition to the explicit test of the null hypothesis), and we implemented this approach to further compare the two models.

**Figure 8:**
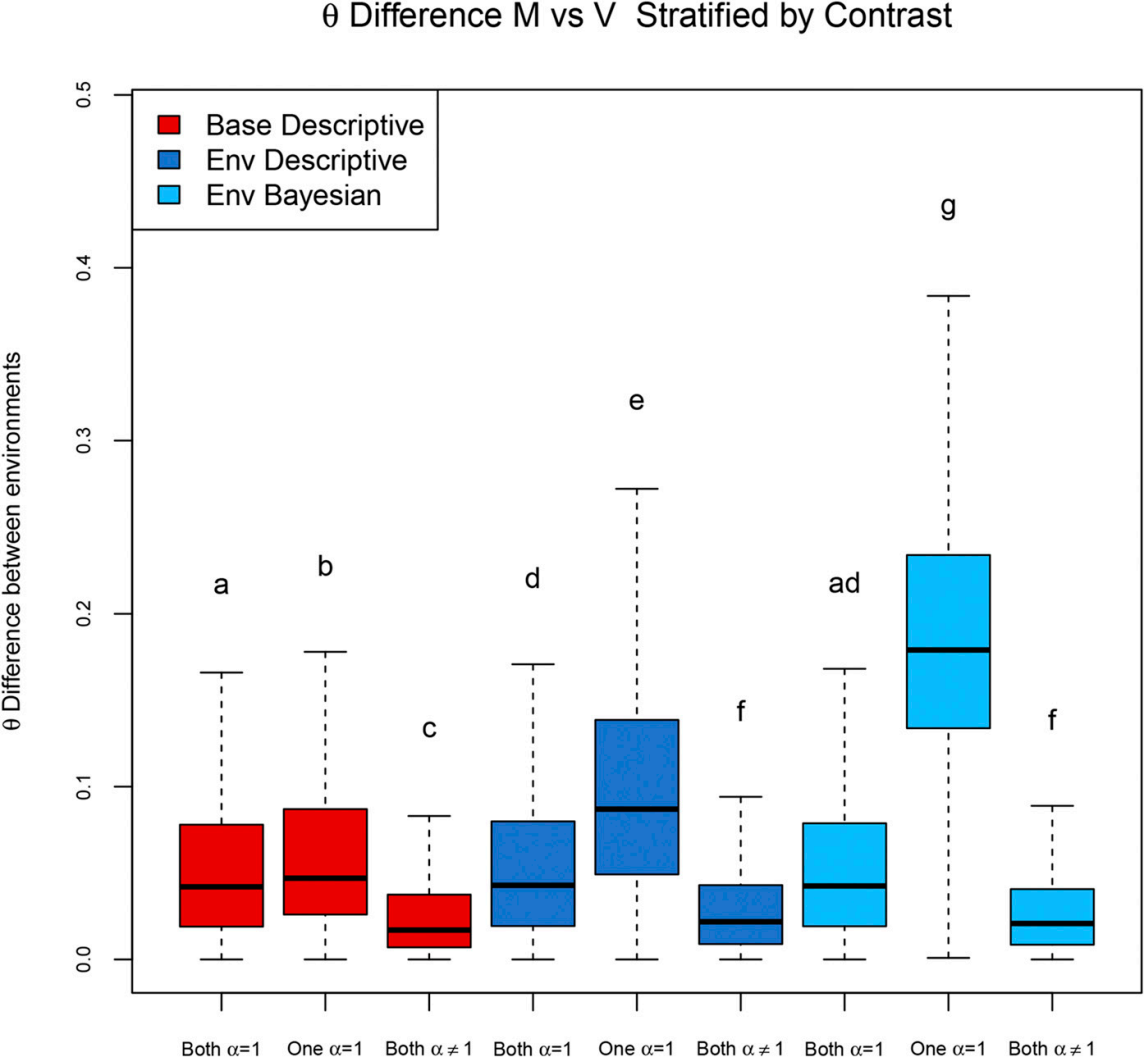
Model comparison. Distribution of absolute difference in ϑ estimates between mated and virgin flies in several contrasts according to different models. Both *α* = 1: AI not detected in mated nor virgin flies. One *α* = 1: AI detected in one mating status alone. Both *α* ≠ 1: AI detected in both mated and virgin flies. Classifications are based on results obtained by the baseline model, for which the difference in AI between environments is reported using a descriptive approach (red), the environmental model, using the descriptive approach for describing difference in AI (dark blue), and the environmental model, using a formal Bayesian test of the null hypothesis for detecting difference in AI (light blue).

Using the baseline model, different AI between environments is declared when AI is present in one of the two environments alone; using the environmental model, it is possible to both identify significantly different levels of AI between environments with a direct test, and indirectly as a comparison between the individual tests within each environment. Using the three possible approaches, we plot in Figure 8 the distribution of ϑ in exons for which no AI was detected (Both *α* = 1), AI was detected in one environment (One *α* = 1), and AI was detected in both environments (Both *α* ≠ 1).

The expectation is that, when the models detected AI in one environment and not in the other (coded here as “One *α* = 1”), the difference in the ϑ distributions is greater than for other situations. This was the case for all the approaches, but the larger difference was observed with the direct test of the null hypothesis *a*1 = *a*2 in the environmental model (Coffman *et al.* 2003).

**Figure 9:**
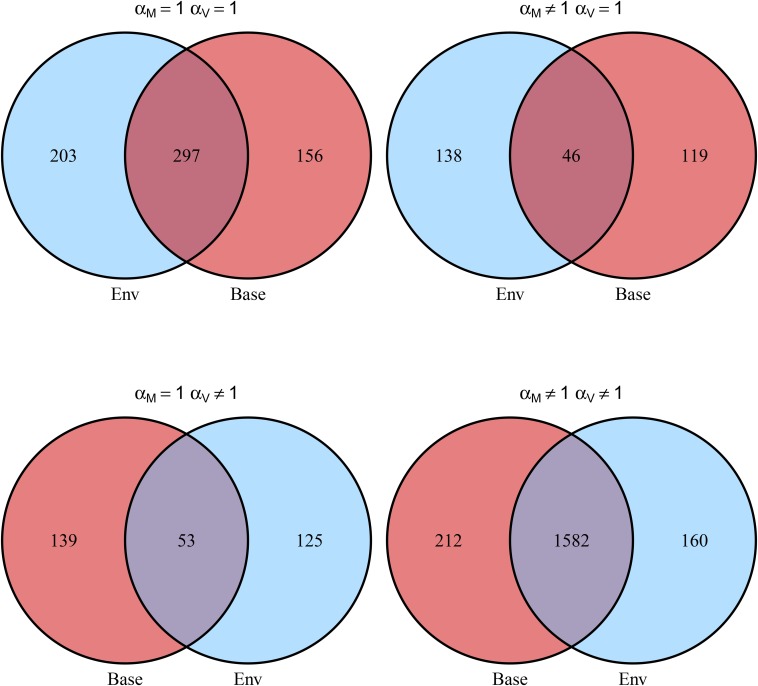
Comparison of AI detected in mated and virgin flies by the two models. Numbers represents the number of genes. A gene was classified as having *α* ≠ 1 when at least one exon in at least one line had *α* ≠ 1.

We counted the number of genes for which at least one exon showed AI in at least one line in mated and virgin flies, respectively; results are shown in Figure 9 and Table 4.

**Table 4:** Comparison of AI detected in mated and virgin flies by the two models

Baseline	*α*_M_ = 1	*α*_M_ ≠ 1
*α*_V_ = 1	500	184
*α*_V_ ≠ 1	178	1742
Environmental	*α*_M_ = 1	*α*_M_ ≠ 1
*α*_V_ = 1	453	165
*α*_V_ ≠ 1	192	1794

*α* = 1: number of genes for which no evidence AI was detected. *α* ≠ 1: number of genes for which at least one exon showed evidence of AI in at least one line. Cohen’s kappa between virgin and mated flies is 0.63 according to the baseline model, and 0.64 according to the environmental model.

#### Analysis of AI in mated and virgin flies using the environmental model:

Analysis of AI using the environmental model was performed on a total of 169,842 exons in lines, belonging to 13,898 different exons in 68 crosses. On average, each exon was analyzed in 12 crosses (range 1–68). The distribution of ϑ in the whole data set is shown in Figure S6 in File S1. As expected, the distribution of ϑ is similar in the two environments—mated and virgin flies—and is centered on 0.5.

**Figure 10:**
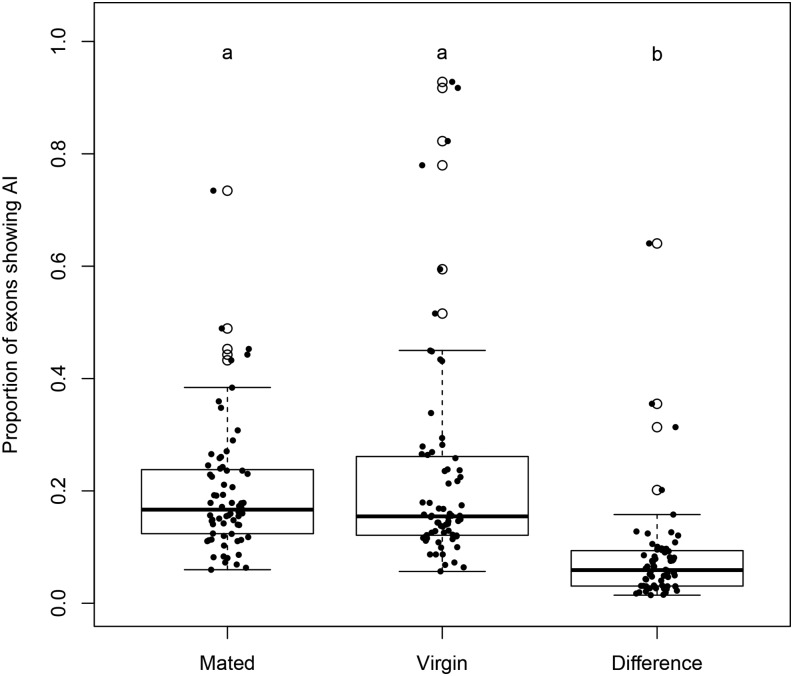
Proportion of exons showing AI. Distribution of proportion of exons showing AI in mated flies, virgin flies, and with different levels of AI between the two mating statuses. Samples that have significantly different distributions are represented by different letters.

Figure 10 shows the distribution of the proportion of exons showing AI in mated and virgin flies, separately, together with the proportion of exons showing a difference in AI between mated and virgin flies. The proportion of exons with a significant difference in AI across environments is significantly lower than the proportion of exons with AI in either one of the environments. This suggests that genetic regulation is generally robust to environmental changes, with the exception of line r149, which is clearly visible as the outlier in the third bar of the boxplot.

To identify genes for which *cis* regulatory variation is either responsive or robust to environmental changes, we list genes that show an excess of AI variation in response to environmental changes, and those that show no variation in AI in response to environmental changes.

We performed Fisher’s exact test to determine if the proportion of lines showing AI in a given exon was significantly higher (Table 5) or lower (Table 6) than the proportion of lines showing AI for all other exons. While the environmental approach does not depend on population frequency of variants, this further analysis does. By searching for excess (or depletion) of differences in AI across environments in the study population, our power depends on the number of exons that can be tested in the population, and this number is smaller for rare variants. This is especially true for Table 6, where a significant depletion of AI across environments can be detected only when a large number of exons (50 or more) have been tested, and none show differences of AI across environments.

**Table 5:** List of the 20 exons showing the highest proportion of differences in AI between mating statuses

Gene*^a^*	Chr*^b^*	Exon Start*^c^*	Exon End*^d^*	Count*^e^*	AI Diff*^f^*	P Diff*^g^*
CG4757	3R	6,986,929	6,987,965	15	7	3.10E−05
pn|Nmd	X	2,075,449	2,077,582	6	4	0.0003
CG10576	3L	5,756,655	5,756,876	16	6	0.0005
CG10924	2R	14,420,177	14,423,254	16	6	0.0005
Vps4	X	17,802,841	17,803,702	22	7	0.0005
CG8920	2R	16,209,183	16,211,729	52	11	0.0008
Gbs-76A	3L	19,275,028	19,278,200	12	5	0.0009
CG14478	2R	13,349,499	13,353,085	53	11	0.0009
CG15465	X	5,068,930	5,070,541	53	11	0.0009
Fur2	X	16,268,959	16,269,892	31	8	0.0010
CG9170	X	15,872,136	15,875,705	62	12	0.0011
CG1910	3R	27,575,303	27,576,308	47	10	0.0013
CG31650	2L	5,042,491	5,042,890	32	8	0.0013
Tomb	2L	5,535,365	5,537,732	4	3	0.0013
CG6404	3L	10,877,834	10,878,890	8	4	0.0013
l(1)G0196	X	21,907,441	21,908,017	34	8	0.0019
CG10932	X	7,781,300	7,782,785	9	4	0.0022
Irc	3R	12,831,146	12,831,711	9	4	0.0022
Mapmodulin	2R	13,753,683	13,754,758	15	5	0.0027
CG8258	2R	4,792,349	4,793,083	5	3	0.0030

a FlyBase gene name.

b Chromosome.

c Exon start position.

d Exon end position.

e Total number of lines in which the exon was tested.

f Total number of lines in which the studied exon showed different levels of AI between mated and virgin flies.

g Fisher’s exact test p-value for excess of lines showing different levels of AI between mated and virgin flies for the studied exon.

**Table 6:** List of the 20 exons showing the least proportion of differences in AI between mating statuses

Gene*^a^*	Chr*^b^*	Exon Start*^c^*	Exon End*^d^*	Count*^e^*	AI Diff*^f^*	P Diff*^g^*
Rack1	2L	7,826,534	7,826,981	68	0	0.007
CG13868	2R	16,196,282	16,197,251	66	0	0.009
eIF-4a	2L	5,985,026	5,985,793	63	0	0.011
HmgZ	2R	17,584,993	17,585,809	63	0	0.011
RpS24	2R	18,528,031	18,528,610	61	0	0.012
Neb-cGP|nocte	X	10,355,115	10,359,100	58	0	0.015
CG5210	2R	12,579,123	12,579,404	57	0	0.016
Gpdh	2L	5,947,240	5,948,843	57	0	0.016
GstE9|imd	2R	14,296,553	14,297,838	57	0	0.016
CG3198	X	6,562,736	6,566,212	56	0	0.018
CG4577	2L	1,139,829	1,141,156	55	0	0.019
CG7378	X	18,802,917	18,805,876	55	0	0.019
CG9140	2L	6,062,079	6,062,435	54	0	0.020
PGRP-LF|UGP	3L	9,343,967	9,344,950	54	0	0.020
Tsp42Ee	2R	2,905,195	2,905,867	54	0	0.020
wupA	X	18,000,714	18,000,855	53	0	0.022
CG15209|CG32669	X	10,736,188	10,739,114	52	0	0.023
CG11073	2R	17,979,647	17,982,292	50	0	0.027
Emc	3L	752,272	753,492	50	0	0.027
l(1)G0156	X	19,412,461	19,413,658	50	0	0.027

a FlyBase gene name.

b Chromosome.

c Exon start position.

d Exon end position.

e Total number of lines in which the exon was tested.

f Total number of lines in which the studied exon showed different levels of AI between mated and virgin flies.

g Fisher’s exact test p-value for depletion of lines showing different levels of AI between mated and virgin flies for the studied exon.

Table 5 lists the 20 genes with the highest excess of lines showing differences in AI between mated and virgin flies, according to Fisher’s exact test. These are the genes that show the highest levels of *cis* by environment variation, which suggests regulatory genetic variation in the response to mating itself.

Table 6 lists the 20 genes showing the least difference in AI between mated and virgin flies, according to Fisher’s exact test. These are genes that show unusually low levels of *cis* by environment variation, compared to other genes in the genome, suggesting robust *cis* activity. Another possible interpretation is that the allele frequency for different regulatory alleles in this population is low.

The environmental model can test variation of AI across environments, genotypes, sex, and other conditions. We compared AI levels between the F1’s r365×w1118 and r907×w1118—the two genotypes having, respectively, the lower and higher proportion of exons with AI. A total of 406 exons was analyzed in both lines and used for this analysis. In mated flies, AI was detected in 16 exons in line r365 and 124 in line r907, while in virgin flies, AI was detected in 14 exons in line r365 and 123 in line r907. Difference in AI between r365 and r907 was detected in 88 and 83 exons, respectively, or ∼20%, indicating a large number of differences between alleles in these two F1s.

### Conclusions

We present a direct approach for simultaneously testing of AI and differences in AI across environments. We measured performance using simulated data, showing that the method has relatively low Type I and Type II error rates. Our model also accounts for differences in magnitude of gene expression counts and unassigned reads, while providing a more conservative method to estimate AI differences between different environments. Our model protects from increases in type I error even when the number of sequenced reads in the two environments differ by 10-fold. We reanalyzed published data to further investigate robustness of AI to environmental conditions. Our results indicate that gene regulation is substantially robust to environmental changes, with a small number of notable exceptions among genes whose expression is affected by mating status. An analysis directly comparing two different F1s shows ∼20% of the exons have differences in *cis* regulation among lines; this estimate is on par with the detection of *cis* differences in regulation in the population, and demonstrates that the environmental model can be used to test G×G across F1s as well as G×E effects.

## Supplementary Material

Supplemental material is available online at www.g3journal.org/lookup/suppl/doi:10.1534/g3.117.300139/-/DC1.
