# Supplementary material for: Direct Testing for Allele-Specific Expression Differences Between Conditions
Source: G3 (Bethesda). 2017 Nov 22;8(2):447–60. doi: 10.1534/g3.117.300139 (PMC5919738; doi:10.1534/g3.117.300139)
Supplement: Supplementary file 1 [file 447FileS1.docx]

# Direct testing for allele-specific expression differences between conditions

Luis Leon-Novelo^1^, Alison R. Gerken^2^, Rita M. Graze^3^, Lauren M. McIntyre^4^, Fabio Marroni ^5,6^

^1^ Department of Biostatistics and Data Science, The University of Texas Health Science Center at Houston-School of Public Health, Houston, TX

^2^ USDA, Agricultural Research Service, Center for Grain and Animal Health Research, 1515 College Avenue, Manhattan, KS

^3^ Department of Biological Sciences, Auburn University, Auburn, AL

^4^ Genetics Institute and Department of Molecular Genetics and Microbiology, University of Florida, Gainesville, FL

^5^ Dipartimento di Scienze Agroalimentari, Ambientali e Animali, Università di Udine, Udine, Italy

^6^ Istituto di Genomica Applicata, Udine, Italy

## Supplementary Methods

### Drosophila culture

All the used lines (DGRP lines, Winter’s lines and the w[1118] strain) were cured of Wolbachia by three generations culture with doxycycline, followed by more than 3 generations of culture in absence of doxycycline prior to use. Fly population densities were controlled when culturing the flies by collecting eggs from population cages and pipetting equal number of eggs (32ul, or ~300 eggs) into culture bottles. Virgins of the DGRP and Winter’s strains were crossed to w[1118] males to generate hybrid progeny (cross #1). The hybrid flies were collected on 15% yeast/sucrose media. On day 5, virgin females were separated into two groups and one group was exposed to w[1118] males (cross #2) for 24 hours. Both groups (mated and virgin) flies were anesthetized using humidified CO2 gas, and in the mated group the females were separated from the males, whereas the virgin controls were mock-sorted using humidified CO2 gas. All flies were placed in plastic snap-cap tubes and the tubes placed in liquid nitrogen.

This note was retrieved from: https://github.com/McIntyre-Lab/papers/tree/master/lehmann_2015/original_data

## Supplementary Figures


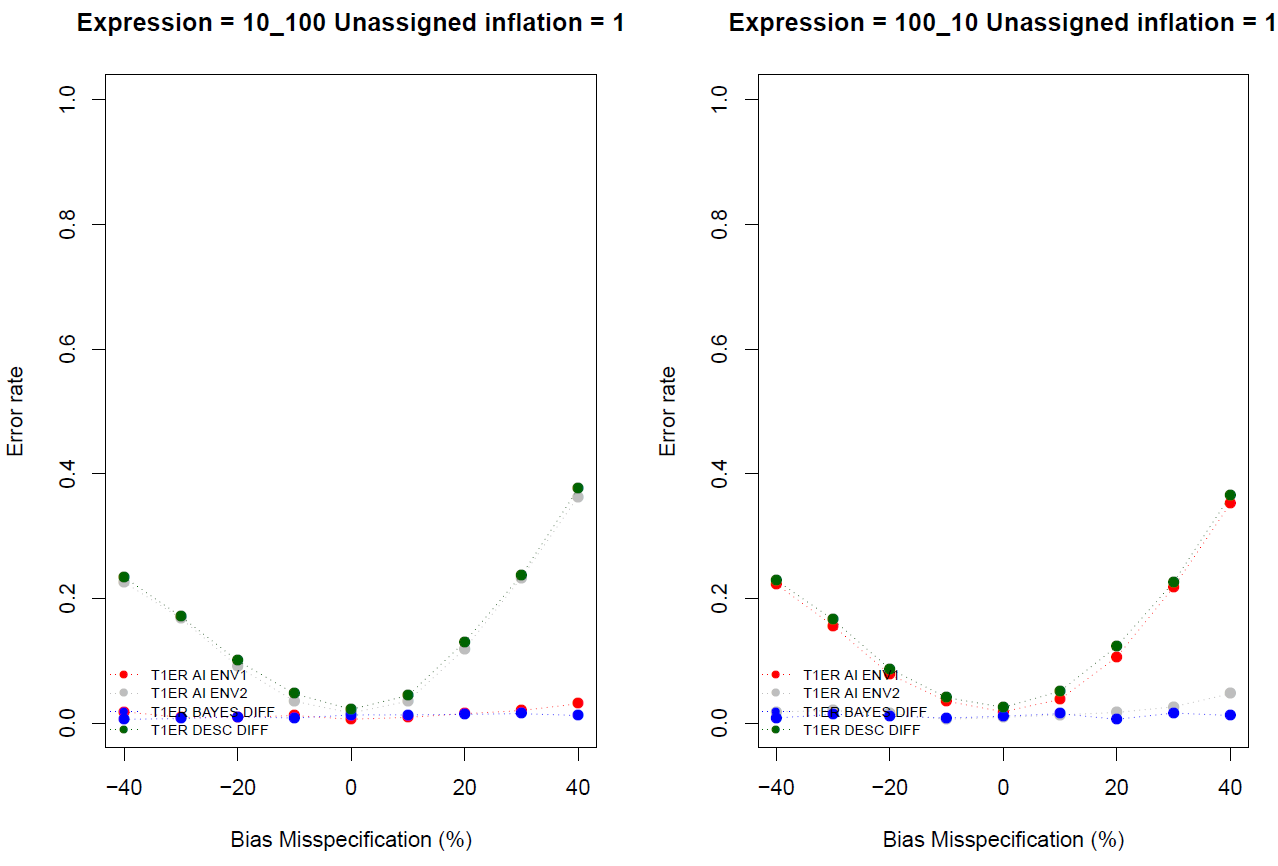


**Figure S1:** Type I error rate in case of low gene expression in Env_1 and high gene expression in Env_2 (left panel), and in case of high gene expression in Env_1 and low gene expression in Env_2 (right panel).


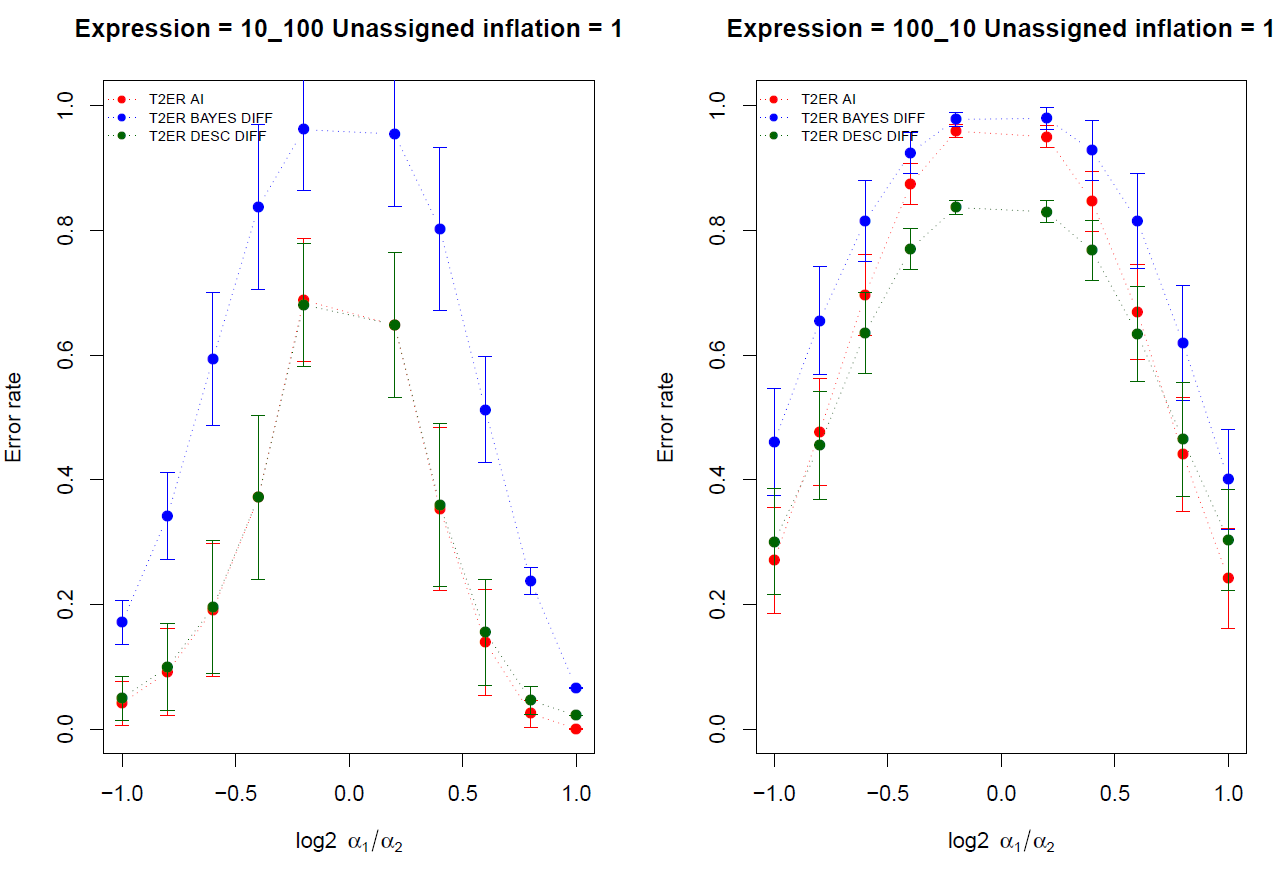


**Figure S2:** Type II error rate (y axis) when comparing an environment for which no AI was present (α_1_ =1) with an environment with varying levels of AI. Differences in AI between environments are reported as log2 α_1_/α_2_. The left panel show type II error rate in case of low gene expression in Env_1 and high gene expression in Env_2. The right panel show type II error rate in case of high gene expression in Env_1 and low gene expression in Env_2. We plot in red the type II error rate in environment 1, in blue the type II error rate for difference in AI between environments and in green the type II error rate between the environments when using the descriptive method. Each point represents the average over the seven simulated levels of bias misspecification, and the error bars represent standard error.


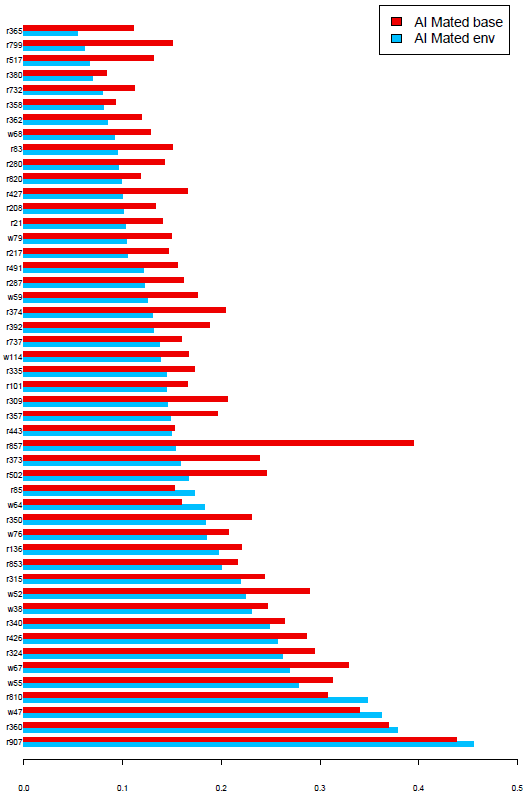


**Figure S3**: Proportion of exons with AI in mated lines according to the environmental (blue) and baseline (red) model, respectively.


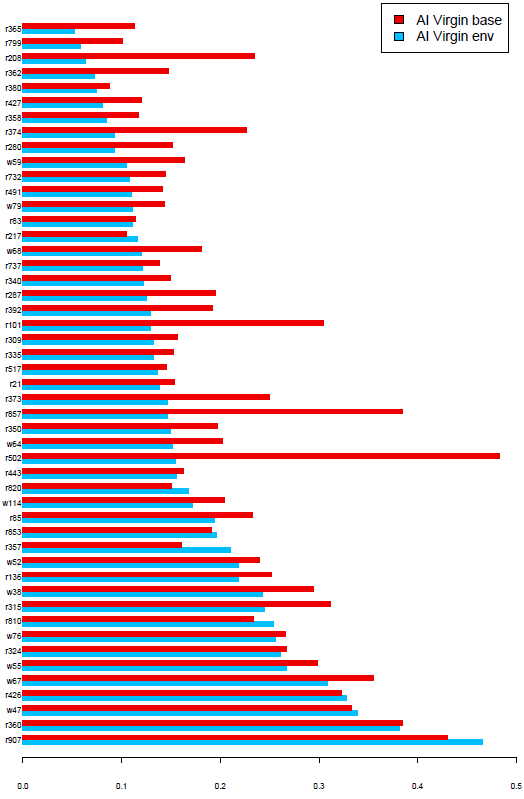


**Figure S4**: Proportion of exons with AI in virgin flies according to the environmental (blue) and baseline (red) model, respectively.


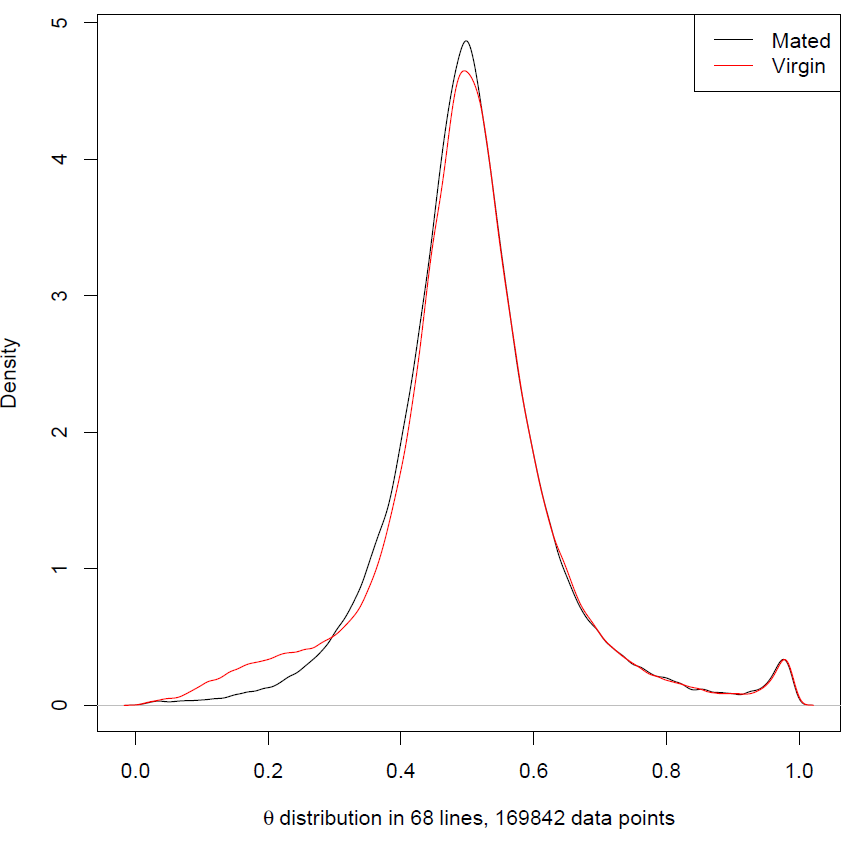


**Figure S5:** Distribution of ϑ in the exons of mated (black line) and virgin (red line) flies.

## Supplementary Tables

**Table S1:** Comparison of results of baseline and environmental model.

|  | **AI across conditions** | **Outside 95% CI** | **Inside 95% CI** | **p-value** |
| --- | --- | --- | --- | --- |
| **Mated** | Base = Env | 478 | 9757 |  |
|  | Base ≠ Env | 1888 | 50325 | 7.01E-07 |
|  | Base ≠ 0 & Env = 0 | 434 | 8658 |  |
|  | (Base ≠ 0 & Env ≠ 0) \| (Base = 0) | 1932 | 51424 | 2.62E-07 |
|  | Env ≠ 0 & Base = 0 | 44 | 1099 |  |
|  | (Env ≠ 0 & Base ≠ 0) \| (Env = 0) | 2322 | 58983 | 0.8758 |
| **Virgin** | Base = Env | 491 | 9744 |  |
|  | Base ≠ Env | 1971 | 50242 | 2.27E-06 |
|  | Base ≠ 0 & Env = 0 | 434 | 8658 |  |
|  | (Base ≠ 0 & Env ≠ 0) \| (Base = 0) | 2028 | 51328 | 1.79E-05 |
|  | Env ≠ 0 & Base = 0 | 57 | 1086 |  |
|  | (Env ≠ 0 & Base ≠ 0) \| (Env = 0) | 2405 | 58900 | 0.07696 |

**Outside 95% CI**: data points for which the difference in estimate of ϑ between the models are outside of the 95% CI estimate. **Inside 95% CI**: data points for which the difference in estimate of ϑ between the models are inside the 95% CI estimate. **Base = Env**: data points for which the baseline and the environmental model give the same estimate for the existence of AI across conditions (both detect no difference, or both detect difference). **Base ≠ Env**: data points for which the baseline and the environmental model give different estimate for the existence of AI across conditions. **Base ≠ 0 & Env = 0**: Baseline model detects difference of AI between conditions and environmental model doesn’t. **(Base ≠ 0 & Env ≠ 0) | (Base = 0)**: Both baseline AND environmental models detect differences, OR baseline model does not detect difference. **Env ≠ 0 & Base = 0**: Environmental model detects difference of AI between conditions and baseline model doesn’t. **(Env ≠ 0 & Base ≠ 0) | (Env = 0)**: Both baseline AND environmental models detect differences, OR environmental model does not detect difference.
